# Impact of indoor residual spraying on malaria parasitaemia in the Bunkpurugu-Yunyoo District in northern Ghana

**DOI:** 10.1186/s13071-018-3130-z

**Published:** 2018-10-23

**Authors:** Benjamin Abuaku, Collins Ahorlu, Paul Psychas, Philip Ricks, Samuel Oppong, Sedzro Mensah, William Sackey, Kwadwo A Koram

**Affiliations:** 1 Epidemiology Department, Noguchi Memorial Institute for Medical Research, College of Health Sciences, University of Ghana, Legon, P. O. Box LG581, Legon, Ghana; 2 University of Florida, 410 NE Waldo Rd, Gainesville, FL 32641 USA; 3 President’s Malaria Initiative/Malaria Branch, Centers for Disease Control and Prevention, Atlanta, GA USA; 4 National Malaria Control Programme, Public Health Division, Ghana Health Service, Accra, Ghana

**Keywords:** Indoor residual spraying, Malaria parasitaemia, Northern Ghana

## Abstract

**Background:**

Since 2008 indoor residual spraying (IRS) has become one of the interventions for malaria control in Ghana. Key partners in the scale-up of IRS have been the US President’s Malaria Initiative (PMI) and AngloGold Ashanti (AGA). This study was designed to assess the impact of IRS on malaria parasitaemia among children less than 5 years-old in Bunkpurugu-Yunyoo, one of PMI-sponsored districts in northern Ghana, where rates of parasitaemia significantly exceeded the national average.

**Methods:**

Two pre-IRS cross-sectional surveys using microscopy were conducted in November 2010 and April 2011 to provide baseline estimates of malaria parasitaemia for the high and low transmission seasons, respectively. IRS for the entire district was conducted in May/June to coincide with the beginning of the rains. Alpha-cypermethrin was used in 2011 and 2012, and changed to pirimiphos-methyl in 2013 and 2014 following declining susceptibility of local vectors to pyrethroids. Post-IRS cross-sectional surveys were conducted between 2011 and 2014 to provide estimates for the end of high (2011–2014) and the end of low (2012–2013) transmission seasons.

**Results:**

The end of high transmission season prevalence of asexual parasitaemia declined marginally from 52.4% (95% CI: 50.0–54.7%) to 47.7% (95% CI: 45.5–49.9%) following 2 years of IRS with alpha-cypermethrin. Prevalence declined substantially to 20.6% (95% CI: 18.4–22.9%) following one year of IRS with pirimiphos-methyl.

**Conclusions:**

The use of a more efficacious insecticide for IRS can reduce malaria parasitaemia among children less than 5 years-old in northern Ghana.

## Background

Indoor residual spraying (IRS) remains one of the two main vector control interventions for malaria prevention, along with long-lasting insecticidal nets (LLINs). Several studies in sub-Saharan Africa and Asia have shown that IRS is associated with reduced malaria transmission in young children and protects all age groups, particularly in combination with LLINs and other control interventions, such as artemisinin-based combination therapy (ACT) and intermittent preventive treatment of pregnant women (IPTp) [1–12]. Following a scale-up of IRS in Africa, it is estimated that the number of Africans protected by IRS increased from 10 million in 2005 to 124 million in 2013 [2]. However, the proportion of the population at risk protected by IRS declined from 10.5% in 2010 to 5.7% in 2015 following a decline in funding between 2013 and 2015 [13].

In 2008, the Ghana National Strategic Plan for malaria control included a scale-up of IRS to cover at least a third of districts in the country by 2015 [14]. Key partners in the scale-up exercise were the US President’s Malaria Initiative (PMI) and AngloGold Ashanti (AGA) [14]. The US PMI’s IRS program focused on selected districts in the northern savannah zone whilst AGA’s program focused on selected districts in the northern savannah zone as well as the forest zone of the country. To monitor the effectiveness of IRS within PMI catchment districts, a study was designed in 2010 to assess the impact of IRS on malaria parasitaemia in one district in the northern savannah zone, which was about to receive IRS in 2011. Data from that study are presented in this paper.

## Methods

### Study site

**Fig. 1:**
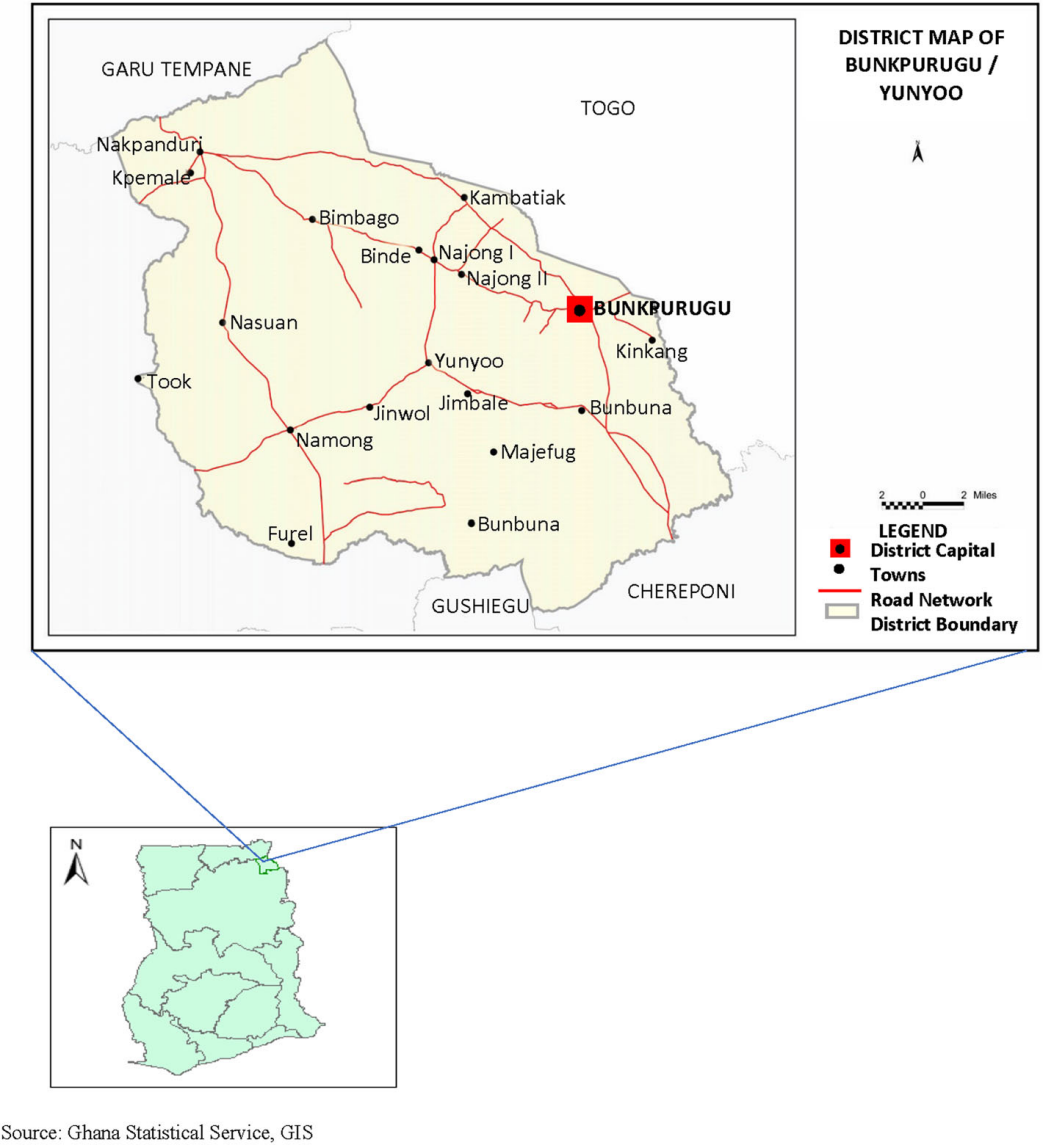
Map of Ghana showing location of Bunkpurugu-Yunyoo District

The study was conducted in the Bunkpurugu-Yunyoo District (10.4846°N, 0.1121°W) located in the northern region of Ghana, where malaria transmission markedly peaks with seasonal rains lasting 3–4 months (August to November) [15]. The district lies within the northern savannah zone with mean annual rainfall of 100–115 mm, and has an estimated population of 122,591, 50.9% of which being female. The district, which is bounded to the east by Togo, west by the East Mamprusi District of Ghana, north by the Garu-Tempane District of Ghana and south by the Gushegu and Chereponi districts of Ghana, is made up of 5 sub-districts: Nasuan, Binde, Nakpanduri, Bunkpurgu and Yunyoo (Fig. 1) [16, 17]. As many as 94.1% of households in the district are engaged in agriculture [16].

The district benefitted from two mass LLIN campaigns implemented by the Ghana Health Service in May 2010 and August 2012. The district was sprayed in 2011 and 2012 using alpha-cypermethrin (0.4% WP) at a rate of 25 mg/m^2^. The declining susceptibility of local vectors [predominantly *Anopheles gambiae* (*s.l.*)] to pyrethroids necessitated the switch of insecticide to pirimiphos-methyl (an organophosophate) at an application rate of 1 g/m^2^ in 2013 and 2014. Generally, IRS operations in the district were conducted in May/June to coincide with the beginning of the rains. In November 2011 and 2012 two of the five sub-districts (Bunkpurugu and Yunyoo) received a second IRS in a design comparing the impact of one-round of IRS per year *vs* two-rounds of IRS per year. This design did not show any superiority of two-round IRS over one-round IRS: malaria prevalence declined from 49.4 to 44.4% in the area with one-round IRS (*P* = 0.037) and from 55.1 to 50.9% in the area with two-round IRS (*P* = 0.071) (unpublished data).

### Study design

**Fig. 2:**
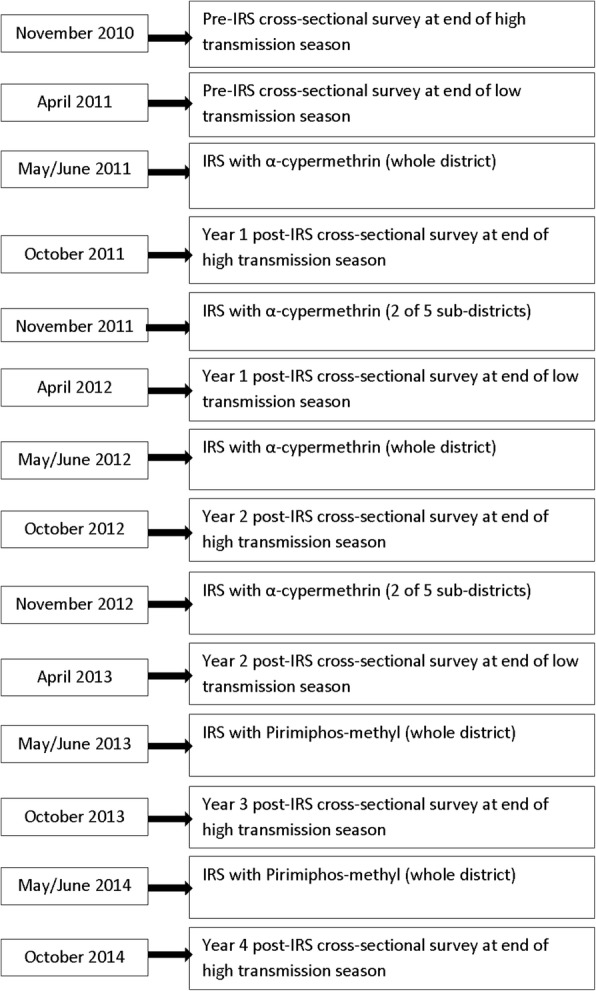
Detailed schedule for IRS activities and cross-sectional surveys in the Bunkpurugu-Yunyoo District (2010–2014)

Two cross-sectional surveys were conducted in November 2010 and April 2011 to provide pre-IRS baseline estimates for the high and low malaria transmission seasons, respectively. Six post-IRS cross-sectional surveys were subsequently conducted between 2011 and 2014 to assess the impact of IRS within the district. Detailed schedules of IRS activities and cross-sectional surveys are shown in Fig. 2. During each survey, probability proportional to size estimates (PPSE) was used to sample 50 communities in the district, followed by a random selection of 17 compounds with children less than five years-old in each selected community. All children less than five years-old within the selected compounds were eligible to participate in the study.

### Data collection

A standard questionnaire was used to obtain data from mothers/caregivers of children less than five years-old in all selected compounds. Data collected included demographic characteristics of mother and child, bednet ownership and use, recent history of fever and recent intake of an antimalarial. Teams comprising an Interviewer, a Prescriber and a laboratory Technician were deployed to specific communities on the basis of the Interviewer’s fluency in the local language. This was to ensure accurate data collection.

### Clinical evaluation

Clinical evaluation by the Prescriber in the team included the measurement of axillary temperature using a digital thermometer (Omron digital, Omron Healthcare Inc., Hoofddorp, the Netherlands). All children were tested for the presence of malaria parasites using the CareStart^TM^ Malaria HRP2/pLDH (pf/PAN) Combo (AccessBio, New Jersey, USA) rapid diagnostic test (mRDT). Children with a history of fever during the 48 h period preceding the day of survey or with an axillary temperature ≥ 37.5 °C at the time of examination and a positive mRDT result were considered as having malaria and treated with artesunate-amodiaquine (AS-AQ) combination as per national treatment guidelines [18]. Children with fever but a negative mRDT result were given an antipyretic and referred to the nearest health centre. Severely ill children were sent to the Baptist Medical Centre, the primary referral hospital in that part of the northern region, for further management at the expense of the project.

### Parasitological evaluation

Samples of blood were obtained through a finger prick, using a sterile lancet, to perform mRDT and prepare thick and thin blood smears for microscopy. The thin smears were fixed in methanol and both thick and thin smears stained with 3% Giemsa stain for 30–45 min. Stained slides were rinsed, dried, and stored in slide boxes for later reading in the research laboratory. For quality control purposes, all blood slides were read by two independent senior microscopists, and discordant slides read by a third senior microscopist. Discordance was related to presence of asexual/sexual parasites as well as plasmodial species identified. For all discordant results, the reading of the third microscopist was considered final. Thick smears were used for parasite quantification whilst thin smears were used for species identification. Parasite quantification was done per 200 white blood cell counts and converted to counts per μl of blood assuming 8000 cells per μl of blood. At least 200 fields of the thick film were examined before declaring a blood slide negative.

### Hematological evaluation

Hemoglobin concentration was determined using a portable automated Hemocue® photometer (Hemocue AB, Ӓngelholm, Sweden). Anaemia was defined as Hb < 11 g/dl using the 2014 Ghana Demographic and Health Survey (GDHS) definitions [19]. Children with Hb < 6 g/dl were considered as having severe anaemia, and were referred to the Baptist Medical Centre.

### Data analysis

The minimum sample size for each survey was 1229, and was based on an estimated prevalence of not more than 60% with 3% precision and 20% non-response rate. Data entries for all surveys were validated by two independent operators using Epidata 3.1. Analysis of validated data was done using SPSS version 21. The outcome variable was malaria parasitaemia by microscopy among children tested. Explanatory variables were gender, age-group, sleeping under a net the night preceding survey, history of fever 48 h prior to survey, and antimalarial intake two weeks prior to survey. Other explanatory variables were caregiver’s age-group, educational status, and occupation as well as bednet availability in the home. Univariate analyses (Chi-square and Fisher’s exact tests) were used to determine significant associations between parasitaemia and the explanatory variables for the high and low transmission seasons.

All variables showing significant association with parasitaemia were used in a multivariate logistic regression to determine their independent effects on parasitaemia in each transmission season adjusting for gender of child and history of IRS. Variables included in the logistic regression for the high transmission season were gender, history of IRS, child’s age-group (months), caregiver’s age-group (years), caregiver’s education, caregiver’s occupation, child sleeping under net night prior to survey, reported fever 48 h prior to survey, and reported antimalarial intake two weeks prior to survey. Variables included in the logistic regression for the low transmission season were gender, time of survey, child’s age-group (months), caregiver’s age-group (years), caregiver’s education, caregiver’s occupation, child sleeping under net night prior to survey, reported fever 48 h prior to survey, and reported antimalarial intake two weeks prior to survey.

## Results

### General characteristics of children tested

Generally, the male to female ratio of children tested in all surveys was approximately 1:1, with approximately 20% of the children aged less than 12 months. In all surveys, over 50% of caregivers of the children tested were 25–34 years-old, had never been to school and practiced farming as an occupation (Tables 1 and 2). Pre-IRS bednet availability in homes visited at the end of the high transmission season was 98.5%, and ranged between 91.1–98.6% during post-IRS surveys in the same season; pre-IRS bednet use among children tested was 95%, and ranged between 82.2–93.7% during post-IRS surveys following application of a pyrethroid, and between 68.1–68.5% following application of an organophosphate (Table 1). At the end of the low transmission season pre-IRS bednet availability was 96.6%, and ranged between 86.9–97.4% during post-IRS surveys following application of a pyrethroid; pre-IRS bednet use among children tested was 62.6% and post-IRS bednet use ranged between 47.7–69.7% (Table 2). The proportion of children who were reported to have had fever within 48 h prior to the pre-IRS survey at the end of the high transmission season was 47.0%, and ranged between 37.3–40.9% during post-IRS surveys following application of a pyrethroid and between 21.3–25.0% during post-IRS surveys following application with an organophosphate (Table 1). The proportion of children who were reported to have had fever within 48 h prior to the pre-IRS survey at the end of the low transmission season was 23.9%, and ranged between 18.7–25.2% during post-IRS surveys following application of a pyrethroid (Table 2). The proportion of children reported to have taken an antimalarial within two weeks prior to each survey conducted at the end of the high transmission season ranged between 2.4–13.8% (Table 1). The proportion of children with anaemia (Hb < 11 g/dl) at the end of the high transmission season pre-IRS survey was 77.7%, and ranged between 67.8–72.5% during post-IRS surveys following application with a pyrethroid, and between 48.3–57.0% during post-IRS surveys following application with an organophosphate (Table 1).

**Table 1:** Background characteristics of children tested in Bunkpurugu-Yunyoo District at the end of the high malaria transmission season (2010–2014)

Characteristic	November 2010 (*n* = 1919)^a^		October 2011 (*n* = 2040)^b^		October 2012 (*n* = 2026)^b^		October 2013 (*n* = 1311)^c^		October 2014 (*n* = 1408)^c^	
	%	95% CI	%	95% CI	%	95% CI	%	95% CI	%	95% CI
Gender										
Male	51.2	48.9–53.5	51.4	49.2–53.6	49.8	47.6–52.0	51.0	48.3–53.7	51.3	48.7–53.9
Female	48.8	46.5–51.1	48.6	46.4–50.8	50.2	48.0–52.4	49.0	46.3–51.7	48.7	46.1–51.4
Age-group (months)										
< 12	19.6	17.9–21.5	20.8	19.1–22.6	20.5	18.8–22.3	22.0	19.8–24.4	21.6	19.5–23.9
12–23	18.6	16.9–20.4	19.6	17.9–21.4	22.4	20.6–24.3	21.6	19.4–24.0	20.5	18.4–22.7
24–35	20.4	18.6–22.3	20.3	18.6–22.1	19.3	17.6–21.1	19.1	17.0–21.4	22.5	20.4–24.8
36–47	20.8	19.0–22.7	19.1	17.4–20.9	18.4	16.8–20.2	18.1	16.1–20.3	20.3	18.3–22.5
48–59	20.6	18.8–22.5	20.2	18.5–22.0	19.3	17.6–21.1	19.2	17.1–21.5	15.1	13.3–17.1
Caregiver’s age-group (years)										
< 25	16.8	15.0–18.8	19.2	17.5–21.0	20.8	19.1–22.7	19.0	16.9–21.3	19.6	17.6–21.8
25–34	52.9	50.4–55.4	54.2	52.0–56.4	52.5	50.3–54.7	53.1	50.4–55.8	53.8	51.2–56.4
35–44	24.7	22.6–26.9	22.3	20.5–24.2	23.1	21.3–25.0	23.7	21.4–26.1	22.9	20.8–25.2
45 and above	5.7	4.6–7.0	4.3	3.5–5.3	3.6	2.9–4.5	4.2	3.2–5.5	3.6	2.7–4.8
Caregiver’s education										
Never attended school	84.2	82.5–85.8	80.7	78.9–82.4	80.6	78.8–82.3	74.4	71.9–76.7	75.3	72.9–77.5
Ever attended school	15.8	14.2–17.5	19.3	17.6–21.1	19.4	17.7–21.2	25.6	23.3–28.1	24.7	22.5–27.1
Caregiver’s occupation										
Non-farming	15.2	13.6–16.9	21.8	20.0–23.7	17.6	16.0–19.3	21.1	18.9–23.4	20.1	18.1–22.3
Farming	84.8	83.1–86.4	78.2	76.3–80.0	82.4	80.7–84.0	78.9	76.6–81.1	79.9	77.7–82.0
Bednet availability in the home										
None	1.5	1.0–2.2	3.7	2.9–4.7	1.4	1.0–2.0	8.9	7.4–10.6	6.8	5.6–8.3
At least one	98.5	97.8–99.0	96.3	95.3–97.1	98.6	98.0–99.1	91.1	89.4–92.6	93.2	91.7–94.5
Child slept under net night prior to survey										
No	5.0	4.1–6.1	17.8	16.2–19.6	6.3	5.3–7.5	31.5	29.0–34.1	31.9	29.5–34.4
Yes	95.0	93.9–95.9	82.2	80.5–83.8	93.7	92.5–94.7	68.5	65.9–71.0	68.1	65.6–70.5
Reported fever 48 h prior to survey										
No	53.0	50.7–55.3	62.8	60.7–64.9	59.1	56.9–61.3	78.7	76.4–80.9	75.0	72.6–77.2
Yes	47.0	44.8–49.3	37.2	35.1–39.4	40.9	38.8–43.1	21.3	19.1–23.6	25.0	22.8–27.4
Antimalarial intake 2 weeks prior to survey										
No	84.6	82.9–86.2	86.2	84.6–87.6	94.1	93.0–95.1	97.6	96.6–98.4	94.8	93.5–95.9
Yes	15.4	13.9–17.1	13.8	12.4–15.4	5.9	4.9–7.0	2.4	1.6–3.4	5.2	4.1–6.5
Anaemia (Hb <11g/dl)										
No	22.3	20.5–24.3	27.5	25.6–29.5	32.2	30.2–34.3	51.7	49.0, 54.4	43.0	40.4–45.6
Yes	77.7	75.6–79.5	72.5	70.5–74.4	67.8	65.7–69.8	48.3	45.6–51.1	57.0	54.4–59.6

^a^Pre-IRS

^b^Post-IRS with pyrethorid

^c^Post-IRS with organophosphate

**Table 2:** Background characteristics of children tested in Bunkpurugu-Yunyoo District at the end of the low malaria transmission season (2011–2013)

Characteristic	April 2011 (*n* = 1967)^a^		April 2012 (*n* = 1984)^b^		April 2013 (*n* = 1998)^b^	
	%	95% CI	%	95% CI	%	95% CI
Gender						
Male	52.2	50.0–54.4	50.3	48.1–52.5	50.2	48.0–52.4
Female	47.8	45.6–50.0	49.7	47.5–51.9	49.8	47.6–52.0
Age-group (months)						
< 12	17.9	16.2–19.7	18.3	16.6–20.1	20.4	18.7–22.3
12–23	20.6	18.9–22.5	21.6	19.8–23.5	22.2	20.4–24.1
24–35	22.2	20.4–24.1	20.3	18.6–22.2	21.1	19.3–23.0
36–47	20.3	18.6–22.2	20.3	18.6–22.2	17.7	16.1–19.5
48–59	19.1	17.4–20.9	19.5	17.8–21.3	18.7	17.0–20.5
Caregiver’s age-group (years)						
< 25	19.2	17.4–21.1	14.4	12.9–16.1	20.4	18.7–22.3
25–34	53.0	50.7–55.3	52.2	50.0–54.4	54.5	52.3–56.7
35–44	24.1	22.2–26.2	28.7	26.7–30.8	22.2	20.4–24.1
45 and above	3.7	2.9–4.7	4.6	3.7–5.7	2.9	2.2–3.8
Caregiver’s education						
Never attended school	83.0	81.3–84.6	83.2	81.5–84.8	77.9	76.0–79.7
Ever attended school	17.0	15.4–18.8	16.8	15.2–18.5	22.1	20.3–24.0
Caregiver’s occupation						
Non-farming	17.1	15.5–18.9	22.4	20.6–24.3	21.7	19.9–23.6
Farming	82.9	81.1–84.5	77.6	75.7–79.4	78.3	76.4–80.1
Bednet availability in the home						
None	3.4	2.7–4.3	13.1	11.7–14.7	2.6	2.0–3.4
At least one	96.6	95.7–97.4	86.9	85.3–88.3	97.4	96.6–98.0
Child slept under net night prior to survey						
No	37.4	35.2–39.6	52.3	50.1–54.5	30.3	28.3–32.4
Yes	62.6	60.4–64.8	47.7	45.5–49.9	69.7	67.6–71.7
Reported fever 48 h prior to survey						
No	76.1	74.1–78.0	74.8	72.8–76.7	81.3	79.5–83.0
Yes	23.9	22.0–25.9	25.2	23.3–27.2	18.7	17.0–20.5
Antimalarial intake 2 weeks prior to survey						
No	96.7	95.8–97.5	97.5	96.7–98.1	98.9	98.4–99.3
Yes	3.3	2.5–4.2	2.5	1.9–3.3	1.1	0.7–1.6
Anaemia (Hb <11g/dl)						
No	57.6	55.3–59.8	53.9	51.7–56.1	53.4	51.2–55.6
Yes	42.4	40.2–44.7	46.1	43.9–48.4	46.6	44.4–48.8

^a^Pre-IRS

^b^Post-IRS with pyrethorid

### Prevalence of parasitaemia among children tested

**Fig. 3:**
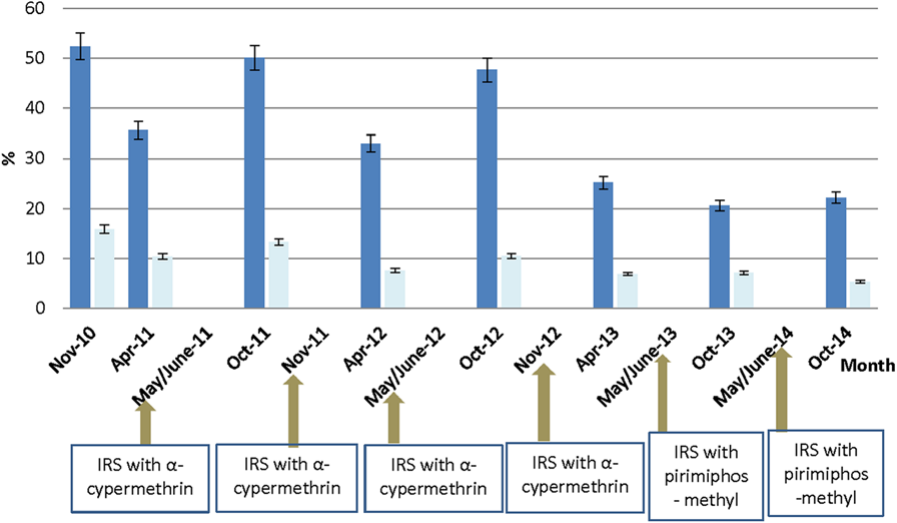
Prevalence of malaria parasitaemia by microscopy in the Bunkpurugu-Yunyoo District. Blue bars represent asexual parasitaemia whilst aqua bars represent gametocytaemia

Prevalence of asexual parasitaemia among children tested at the end of the high transmission season declined from 52.4% (95% CI: 50.0–54.7%) in November 2010 to 47.7% (95% CI: 45.5–49.9%) in October 2012 (a difference of 4.7%; 95% CI: 1.5–10.0%) following two years of alpha-cypermethrin application. Prevalence further declined from 47.7% in October 2012 to 20.6% (95% CI: 18.4–22.9%) in October 2013 (a difference of 27.1%; 95% CI: 24.0–30.3%) following the application of pirimiphos-methyl, and remained stable in October 2014 at 22.2% (95% CI: 20.1–24.5%) following the second year application of pirimiphos-methyl (Fig. 3). Prevalence of asexual parasitaemia among children tested at the end of the low transmission season significantly declined from 35.6% (95% CI: 33.5–37.8%) in April 2011 to 25.2% (95% CI: 23.4–27.2%) in April 2013 (a difference of 10.4%; 95% CI: 7.5–13.3%) following two years of alpha-cypermethrin application (Fig. 3). Prevalence of gametocytaemia at the end of the high transmission season significantly declined from 15.9% (95% CI: 14.2–17.7%) in November 2010 to 5.3% (95% CI: 4.2–6.6%) in October 2014 (a difference of 10.6%; 95% CI: 8.5–12.7%) following two years of alpha-cypermethrin application and two years of organophosphate application. Gametocytaemia at the end of the low transmission season significantly declined from 10.4% (95% CI: 9.1–11.9%) in April 2011 to 6.9% (95% CI: 5.9–8.1%) in April 2013 (a difference of 3.5%; 95% CI: 1.7–5.3%) following two years of alpha-cypermethrin application (Fig. 3). Most infections were *Plasmodium falciparum*, either alone (87–96.5% in surveys conducted at the end of the high transmission season and 83.1–87.7% in surveys conducted at the end of the low transmission season), mixed with *Plasmosium malariae* (1.5–4.9% in surveys conducted at the end of the high transmission season and 6.1–9.7% in surveys conducted at the end of the low transmission season) or mixed with *Plasmodium ovale* (0.2–2.3% in surveys conducted at the end of the high transmission season and 0–0.4% in surveys conducted at the end of the low transmission season). Geometric mean parasite density ranged between 2022–4373/μl at the end of the high transmission season, and between 1577–2230/μl at the end of the low transmission season.

### Univariate analyses of indicators associated with malaria parasitaemia

Univariate analyses showed that the prevalence of malaria parasitaemia at the end of both high and low transmission seasons was significantly associated with: (i) age of child tested; (ii) age, educational status, and occupation of caregiver; (iii) child reported to have had fever within 48 h prior to survey; and (iv) child reported to have taken an antimalarial within two weeks prior to survey (Table 3). Additionally, parasitaemia at the end of the high transmission season was significantly associated with child sleeping under a net the night prior to survey (Table 3).

**Table 3:** Association between selected indicators and malaria asexual parasitaemia at the end of the high and low transmission seasons in Bunkpurugu-Yunyoo District (2010–2014)

Characteristic	High season			Low season		
	Total	%	*P*-value	Total	%	*P*-value
Gender						
Male	4323	40.6	0.825	3008	31.8	0.312
Female	4158	40.9		2900	30.6	
Age-group (months)						
< 12	1767	23.4	<0.001	1113	8.4	<0.001
12–23	1754	37.3		1268	23.5	
24–35	1716	43.5		1257	34.8	
36–47	1642	49.2		1144	43.1	
48–59	1615	52.0		1127	46.4	
Caregiver’s age-group (years)						
< 25	1569	35.1	<0.001	1032	25.9	<0.001
25–34	4389	39.0		3055	29.2	
35–44	1901	45.9		1436	36.2	
45 and above	348	44.8		215	35.3	
Caregiver’s education						
Never attended school	6770	45.7	<0.001	4810	35.3	<0.001
Ever attended school	1742	22.2		1103	13.5	
Caregiver’s occupation						
Non-farming	1614	26.0	<0.001	1200	16.5	<0.001
Farming	6866	44.3		4705	34.9	
Child slept under net night prior to survey						
No	1432	34.8	<0.001	2340	31.7	0.545
Yes	7020	42.0		3511	30.9	
Reported fever 48 h prior to survey						
No	5467	30.4	<0.001	4532	24.1	<0.001
Yes	3020	59.8		1323	55.4	
Antimalarial intake 2 weeks prior to survey						
No	7741	42.0	<0.001	5779	31.6	<0.001
Yes	772	29.5		134	16.4	

Prevalence of malaria parasitaemia at the end of the high transmission season was: (i) significantly increased with increasing age of child (from 23.4% among children aged less than 12 months to 52.0% among children aged 48–59 months); (ii) increased with increasing age of caregiver (from 35.1% among caregivers aged less than 25 years to 44.8% among caregivers aged 45 years and above); (iii) higher among caregivers who never attended school compared with those who attended school (45.7 *vs* 22.2%); (iv) higher among caregivers who were farmers compared with non-farmers (44.3 *vs* 26.0%); (v) higher among children who were reported to have slept under a net night preceding the survey compared with those who did not sleep under a net (42.0 *vs* 34.8%); (vi) higher among children reported to have had fever 48 h prior to survey compared with those without fever (59.8 *vs* 30.4%); and (vii) higher among children reported not to have taken an antimalarial two weeks prior to survey compared with those who had taken an antimalarial (42.0 *vs* 29.5%) (Table 3).

Prevalence of malaria parasitaemia at the end of the low transmission season was: (i) significantly increased with increasing age of child (from 8.4% among children aged less than 12 months to 46.4% among children aged 48–59 months); (ii) increased with increasing age of caregiver (from 25.9% among caregivers aged less than 25 years to 35.3% among caregivers aged 45 years and above); (iii) higher among caregivers who never attended school compared with those who attended school (35.3 *vs* 13.5%); higher among caregivers who were farmers compared with non-farmers (34.9 *vs* 16.5%); (iv) higher among children reported to have had fever 48 h prior to survey compared with those without fever (55.4 *vs* 24.1%); and (v) higher among children reported not to have taken an antimalarial two weeks prior to survey compared with those who had taken an antimalarial (31.6 *vs* 16.4%) (Table 3).

### Multivariate analyses of indicators associated with malaria parasitaemia

When all indicators significantly associated with asexual parasitaemia in the univariate analyses were included in a logistic regression analysis, adjusting for gender and history of IRS, asexual parasitaemia was not associated with caregiver’s age-group or child sleeping under a net night prior to survey at the end of either transmission season. The odds of parasitaemia significantly increased with age in both transmission seasons: (i) from 1.9 (95% CI: 1.6–2.2) among children aged 12–23 months to 3.5 (95% CI: 2.9–4.1) among children aged 48–59 months at the end of the high transmission season; and (ii) from 3.3 (95% CI: 2.5–4.3) among children aged 12–23 months to 10.7 (95% CI: 8.2–13.9) among children aged 48–59 months at the end of the low transmission season (Table 4). Odds of parasitaemia at the end of both transmission seasons were significantly lower among caregivers who indicated they had ever attended school compared with those who had never attended school (OR = 0.4, 95% CI: 0.4–0.5 for the high transmission season and OR = 0.4, 95% CI: 0.3–0.5 for the low transmission season). Caregivers who were farmers had higher odds of parasitaemia compared with non-farmers for both transmission seasons (OR = 1.7, 95% CI: 1.5–2.0 for the high transmission season and OR = 2.2, 95% CI: 1.8–2.7 for the low transmission season). Odds of parasitaemia at the end of both transmission seasons were significantly higher among children who were reported to have had fever within 48 h prior to survey compared with those who had no fever (OR = 3.2, 95% CI: 2.9–3.6 for the high transmission season and OR = 4.4, 95% CI: 3.8–5.1 for the low transmission season). Children who were reported to have taken an antimalarial within two weeks prior to survey had lower odds of parasitaemia compared with those who had not taken any antimalarial (OR = 0.3, 95% CI: 0.3–0.4 for the high season and OR = 0.3, 95% CI: 0.2–0.5 for the low season) (Table 4). History of IRS was significantly associated with parasitaemia in both transmission seasons. The association observed in the high transmission season was not evident until the third year when pyrethroid was replaced with organophosphate. The odds of parasitaemia was significantly lower after the application of an organophosphate compared with the pre-IRS survey (OR = 0.3, 95% CI: 0.2–0.3), and remained stable after another round of IRS with an organophosphate (OR = 0.3, 95% CI: 0.2–0.3) (Table 4).

**Table 4:** Multivariate logistic regression analysis of indicators associated with malaria asexual parasitaemia by transmission season in Bunkpurugu-Yunyoo District

Characteristic	High season			Low season		
	OR	95% CI	*P*-value	OR	95% CI	*P-*value
Gender						
Male^a^						
Female	1.1	1.0–1.2	0.070	1.0	0.8–1.1	0.521
History of IRS (high season)						
None^a^						
Year 1 IRS with α-cypermethrin	1.1	0.9–1.3	0.190	–	–	–
Year 2 IRS with α-cypermethrin	0.9	0.7–1.0	0.058	–	–	–
Year 3 IRS with pirimiphos-methyl	0.3	0.2–0.3	<0.001	–	–	–
Year 4 IRS with pirimiphos-methyl	0.3	0.2–0.3	<0.001	–	–	–
History of IRS (low season)						
None^a^						
Year 1 IRS with α-cypermethrin	–	–	–	0.9	0.8–1.1	0.352
Year 2 IRS with α-cypermethrin	–	–	–	0.7	0.6–0.8	<0.001
Age-group (months)						
< 12^a^						
12–23	1.9	1.6–2.2	<0.001	3.3	2.5–4.3	<0.001
24–35	2.4	2.0–2.8	<0.001	6.1	4.7–8.0	<0.001
36–47	3.1	2.6–3.6	<0.001	9.3	7.2–12.2	<0.001
48–59	3.5	2.9–4.1	<0.001	10.7	8.2–13.9	<0.001
Caregiver’s age-group (years)						
< 25^a^						
25–34	0.9	0.8–1.0	0.144	0.8	0.7–1.0	0.059
35–44	1.0	0.9–1.2	0.732	0.9	0.8–1.2	0.572
45 and above	0.9	0.7–1.1	0.224	0.8	0.5–1.1	0.130
Caregiver’s education						
Never attended school^a^						
Ever attended school	0.4	0.4–0.5	<0.001	0.4	0.3–0.5	<0.001
Caregiver’s occupation						
Non-farming^a^						
Farming	1.7	1.5–2.0	<0.001	2.2	1.8–2.7	<0.001
Child slept under net night prior to survey						
No^a^						
Yes	0.9	0.8–1.1	0.181	1.0	0.9–1.2	0.864
Reported fever 48 h prior to survey						
No^a^						
Yes	3.2	2.9–3.6	<0.001	4.4	3.8–5.1	<0.001
Antimalarial intake 2 weeks prior to survey						
No^a^						
Yes	0.3	0.3–0.4	<0.001	0.3	0.2–0.5	<0.001

^a^Reference category

## Discussion

A series of cross-sectional surveys conducted between 2010 and 2014 showed a positive impact of IRS on malaria parasitaemia in the Bunkpurugu-Yunyoo District in northern Ghana. Malaria parasitaemia was found to be significantly associated with similar characteristics in both the high and low transmission seasons. The factors that associated with asexual parasitaemia during both transmission seasons were: increasing age of child; reported fever within 48 h prior to survey; reported intake of an antimalarial within two weeks prior to survey; caregiver’s educational status; and caregiver’s occupation.

Prevalence of malaria asexual parasitaemia declined by 9.0 and 29.2% at the end of the high and low transmission seasons, respectively, after 2 years of IRS with alpha-cypermethrin. The quantum of decline does not agree with findings from Sao Tome and Principe, another sub-Saharan African country, where malaria parasitaemia reduced by 97% [from 20.1% (95% CI: 18.0–22.4%) to 0.6% (95% CI: 0.2–1.6%)] after two years of annual IRS with alpha-cypermethrin [20]. Prevalence of malaria asexual parasitaemia, however, declined by almost 57% at the end of the peak transmission season of 2013 after using pirimiphos-methyl. The better performance of pirimiphos-methyl, compared with alpha-cypermethrin, can be explained by the high levels of pyrethroid resistance reported in the district (60–90% mosquito susceptibility to pyrethroids and 98–100% susceptibility to organophosphates) (unpublished 2012 data) and other parts of the country [21, 22]. The decision to change insecticide in Bunkpurugu-Yunyoo District in 2013 was therefore appropriate for achieving better impact on malaria parasitaemia. After the second year application of pirimiphos-methyl in 2014, prevalence of asexual parasitaemia remained stable at 22.2% compared with the 2013 prevalence of 20.6%. This finding of stable malaria prevalence or no further decline in prevalence after two consecutive applications of an alternative insecticide suggests that other interventions will be critical in achieving consistent decline in parasitaemia in areas where IRS is deployed. Although LLIN ownership at the end of the high transmission season in the Bunkpurugu-Yunyoo District ranged between 91.1–98.6% over the 5 year period of this study, net-use declined over the years from 95% to 68.1%. However, high transmission seasons of 2010 and 2012 experienced over 90% LLIN usage rate following mass campaigns. This observation suggests that promoting LLIN use, during the high transmission season in particular, is necessary to benefit from the contributing effect of LLINs on the reduction of malaria prevalence.

Risk of parasitemia increased with increasing age group, irrespective of transmission season. Odds of parasitaemia almost doubled from 1.9 among children aged 12–23 months to 3.5 among children aged 48–59 months at the end of the high transmission season and almost tripled from 3.3 among children aged 12–23 months to 10.7 among children aged 48–59 months at the end of the low transmission season. This finding compares well with findings from the 2011 multiple indicator cluster survey in Ghana, suggesting that older child age-groups within children less than five years bear the highest burden of malaria infection [23]. Malaria interventions for children less than five year-old should therefore be designed to take care of the vulnerabilities of children within the older age-groups, particularly those aged 3 years and above.

As expected, the risk of parasitaemia was higher among children with reported history of fever and children reported not to have taken an antimalarial within two weeks prior to surveys at the end of either transmission season. This finding suggests that fever remains a predictor of malaria infection in the study district, and therefore supports global and national guidelines for case management of malaria [18, 24, 25]. Use of antimalarials is primarily aimed at both parasitological and clinical cure, and therefore a good predictor of absence of parasitaemia, particularly in the era of artemisinin-based combination therapy [24].

For both transmission seasons, the odds of parasitaemia were higher among children with caregivers who had never attended school. This finding compares well with the 2011 multiple indicator survey, which showed that malaria parasitaemia was highest among children with caregivers who had never attended school [23]. Mother’s education has generally been associated with child health inequalities, and so empowering women with education could go a long way to impact on childhood malaria prevalence reduction, among others [26]. Children with caregivers who engaged in farming as an occupation had almost a double odds of parasitaemia compared with children with non-farming caregivers. Farming in Ghana is largely practiced by persons with low levels of education, and therefore it is not surprising to see the two characteristics showing a similar effect on parasitaemia in the Bunkpurugu-Yunyoo District. Also, compound farming, a home garden-type of agroforestry system, is one of the major types of farming practiced in northern Ghana, and so increases exposure to outdoor mosquito bites [27, 28].

Our study has a couple of limitations. Our evidence of a positive impact of IRS on malaria parasitaemia would have been stronger if we had a comparison area. Our study was designed in a period when IRS was being scaled-up in the northern region of Ghana, and therefore there was limited opportunity to have a comparison area. Nevertheless, our study provides useful insight into the impact of IRS on parasitaemia and other indicators such as fever and anaemia. Another limitation is our inability to control for migration; we did not consider movement of persons in and out of the district to be able to estimate different levels of exposure to mosquito vectors. Although we cannot conclude that living within the district adequately protects against malaria infection, several studies have shown that IRS is associated with reduced malaria transmission. Our entomological monitoring in the district over a period of two years showed entomological inoculation rates (EIRs) declining from pre-IRS level of 0.350 infective bites/person/year to post-IRS level of 0.021 infective bites/person/year (unpublished data).

## Conclusions

Following a 2-year application of alpha-cypermethrin at the beginning of the high malaria transmission season in the Bunkpurugu-Yunyoo District, the end of high transmission season prevalence of malaria parasitaemia declined by only 9%, whilst a change of insecticide to pirimiphos-methyl yielded a decline of 57% after one year of application. We conclude that the use of a more efficacious insecticide for IRS can reduce malaria parasitaemia among children less than five years-old in northern Ghana.

## Abbreviations

ACT: Artemisinin-based combination therapy
AGA: AngloGold Ashanti
AS-AQ: Artesunate-amodiaquine combination
IPTp: Intermittent preventive treatment of pregnant women
IRS: Indoor residual spraying
LLINs: Long-lasting insecticidal nets
mRDT: Malaria rapid diagnostic test
PMI: President’s Malaria Initiative

## References

[1] Mabaso ML, Sharp B, Lengeler C (2004). Historical review of malarial control in southern African with emphasis on the use of indoor residual house-spraying. Trop Med Int Health.

[2] WHO. Indoor Residual Spraying: An Operational Manual for Indoor Residual Spraying (IRS) for Malaria Transmission Control and Elimination. 2nd ed. Geneva: World Health Organization; 2015.

[3] Sharp BL, Ridl FC, Govender D, Kuklinski J, Kleinschmidt I (2007). Malaria vector control by indoor residual insecticide spraying on the tropical Island of Bioko, Equatorial Guinea. Malar J.

[4] Misra SP, Webber R, Lines J, Jaffar S, Bradley DJ. Malaria control: bednets or spraying? Spray *versus *treated nets using deltamethrin - a community randomized trial in India. Trans R Soc Trop Med Hyg. 1999;93:456–7. 10.1016/s0035-9203(99)90335-8 10696395

[5] Rowland M, Mahmood P, Igbal J, Carneiro I, Chavasse D (2000). Indoor residual spraying with alphacypermethrin controls malaria in Pakistan: a community randomized trial. Trop Med Int Health.

[6] Curtis CF, Maxwell CA, Finch RJ, Njunwa KJ (1998). A comparison of use of a pyrethroid either for house spraying of for bednet treatment against malaria vectors. Trop Med Int Health.

[7] Kleinschmidt I, Sharp B, Benavente LE, Schwabe C, Torrez M, Kuklinski J (2006). Reduction in infection with *Plasmodium falciparum* one year after the introduction of malaria control interventions on Bioko Island, Equatorial Guinea. Am J Trop Med Hyg.

[8] WHO (2011). World Malaria Report.

[9] Barnes KI, Durrheim DN, Little F, Jackson A, Mehta U, Allen E (2005). Effect of artemether-lumefantrine policy and improved vector control on malaria burden in KwaZulu-Natal, South Africa. PLoS Med.

[10] Kleinschmidt I, Scwabe C, Shiva M, Segura JL, Sima V, Mabunda SJA (2009). Combining indoor residual spraying and insecticide-treated net interventions. Am J Trop Med Hyg.

[11] Korenromp EL, Armstrong-Schellenberg RM, Williams BG, Nahlen BL, Snow RW (2004). Impact of malaria control on childhood anaemia in Africa - a quantitative review. Trop Med Int Health.

[12] Kim D, Fedak K, Kramer R (2012). Reduction of malaria prevalence by indoor residual spraying: a meta-regression analysis. Am J Trop Med Hyg.

[13] WHO (2016). World Malaria Report.

[14] Ministry of Health, Ghana (2009). Strategic Plan for Malaria Control in Ghana 2008–2015.

[15] Baird JK, Owusu Agyei S, Utz GC, Koram K, Barcus MJ, Jones TR (2002). Seasonal malaria attack rates in infants and young children in northern Ghana. Am J Trop Med Hyg.

[16] Ghana Statistical Service. 2010 Population & Housing Census. District Analytical Report. Bunkpurugu Yunyoo District. Accra: Ghana Statistical Service; 2014.

[17] Composite Budget for 2018-2021. Programme based budget Estimates for 2018. Bunkpurugu/Yunyoo District Assembly. https://www.mofep.gov.gh/sites/default/files/composite-budget/2018/NR/Bunkpurugu.pdf. Accessed 10 Oct 2018.

[18] Ministry of Health, Ghana (2014). Guidelines for Case Management of Malaria in Ghana.

[19] Ghana Statistical Service, Ghana Health Service, and ICF International (2015). Ghana Demographic and Health Survey 2014.

[20] Tseng LF, Chang WC, Ferreira MC, Wu CH, Rampao HS, Lien JC (2008). Rapid control of malaria by means of indoor residual spraying of alphacypermethrin in the Democratic Republic of Sao Tome and Principe. Am J Trop Med Hyg.

[21] Baffour-Awuah S, Annan AA, Maiga-Ascofare O, Dieudonné SD, Adjei-Kusi P, Owusu-Dabo E (2016). Insecticide resistance in malaria vectors in Kumasi, Ghana. Parasit Vectors.

[22] Hunt RH, Fuseini G, Knowles S, Stiles-Ocran J, Verster R, Kaiser ML (2011). Insecticide resistance in malaria vector mosquitoes at four localities in Ghana, West Africa. Parasit Vectors.

[23] Ghana Statistical Service (2011). Ghana Multiple Indicator Cluster Survey with an Enhanced Malaria Module and Biomarker, 2011, Final Report.

[24] WHO (2010). Guidelines for the Treatment of Malaria.

[25] Okiro EA, Snow RW (2010). The relationship between reported fever and *Plasmodium falciparum* infection in African children. Malar J.

[26] Wamani H, Tylleskar T, Astrom AN, Tumwine JK, Peterson S (2004). Mothers’ education but not fathers’ education, household assets or land ownership is the best predictor of child health inequalities in Uganda. Int J Equity Health.

[27] Govella NJ, Ferguson H (2012). Why use of interventions targeting outdoor biting mosquitoes will be necessary to achieve malaria elimination. Front Physiol.

[28] Reddy MR, Overgaard HJ, Abaga S, Reddy VP, Caccone A, Kiszewski AE (2011). Outdoor host seeking behavior of *Anopheles gambiae* mosquitoes following initiation of malaria vector control on Bioko Island, Equatorial Guinea. Malar J.

